# Characterization of clostridium botulinum neurotoxin serotype A (BoNT/A) and fibroblast growth factor receptor interactions using novel receptor dimerization assay

**DOI:** 10.1038/s41598-021-87331-7

**Published:** 2021-04-09

**Authors:** Nicholas G. James, Shiazah Malik, Bethany J. Sanstrum, Catherine Rhéaume, Ron S. Broide, David M. Jameson, Amy Brideau-Andersen, Birgitte S. Jacky

**Affiliations:** 1 Department of Cell and Molecular Biology, John A. Burns School of Medicine, University of Hawaii, 651 Ilalo St., BSB 222, Honolulu, HI 96813 USA; 2 Neurotoxin Research Program, Department of Biological Sciences, Allergan (an AbbVie Company) R&D, 2525 Dupont Dr., RD3-3B, Irvine, CA 92612 USA

**Keywords:** Cell biology, Molecular biology, Neuroscience

## Abstract

Clostridium botulinum neurotoxin serotype A (BoNT/A) is a potent neurotoxin that serves as an effective therapeutic for several neuromuscular disorders via induction of temporary muscular paralysis. Specific binding and internalization of BoNT/A into neuronal cells is mediated by its binding domain (H_C_/A), which binds to gangliosides, including GT1b, and protein cell surface receptors, including SV2. Previously, recombinant H_C_/A was also shown to bind to FGFR3. As FGFR dimerization is an indirect measure of ligand-receptor binding, an FCS & TIRF receptor dimerization assay was developed to measure rH_C_/A-induced dimerization of fluorescently tagged FGFR subtypes (FGFR1-3) in cells. rH_C_/A dimerized FGFR subtypes in the rank order FGFR3c (EC_50_ ≈ 27 nM) > FGFR2b (EC_50_ ≈ 70 nM) > FGFR1c (EC_50_ ≈ 163 nM); rH_C_/A dimerized FGFR3c with similar potency as the native FGFR3c ligand, FGF9 (EC_50_ ≈ 18 nM). Mutating the ganglioside binding site in H_C_/A, or removal of GT1b from the media, resulted in decreased dimerization. Interestingly, reduced dimerization was also observed with an SV2 mutant variant of H_C/_A. Overall, the results suggest that the FCS & TIRF receptor dimerization assay can assess FGFR dimerization with known and novel ligands and support a model wherein H_C_/A, either directly or indirectly, interacts with FGFRs and induces receptor dimerization.

## Introduction

Botulinum neurotoxin type A (BoNT/A) is a 150 kDa metalloenzyme belonging to the family of neurotoxins produced by *Clostridium botulinum*. The toxin causes temporary muscle paralysis by inhibiting acetylcholine release at the neuromuscular junction^1–4^. The neuronal specificity and high potency of BoNT/A has allowed its use in the treatment of a large number of medical and aesthetic conditions^3,5–7^, relying on injection of picomolar (pM) concentrations of the toxin. Though BoNT/A has been the subject of extensive study, greater understanding of the complex mechanism associated with BoNT/A’s neuronal specificity and cellular entry could lead to further therapeutic applications.

BoNT/A is a single-chain protein activated by proteolytic cleavage to form a 150 kDa di-chain molecule. The di-chain is composed of a light chain (L_C_/A), which encodes a Zn^2+^-dependent endopeptidase (~ 50 kDa), linked by a single disulfide bond and non-covalent interactions to a ~ 100 kDa heavy chain (HC) containing the receptor binding and translocation domains^8^. The 50 kDa receptor binding domain, H_C_/A, is located at the C-terminal half of the HC and mediates specific binding and internalization of the toxin into neurons. Following internalization, the translocation domain (H_N_) of BoNT/A, residing at the N-terminal half of the HC, facilitates the translocation of L_C_/A from the endocytic vesicle into the cytosol. Once in the cytosol, L_C_/A enzymatically cleaves the soluble N-ethylmaleimide-sensitive factor attachment protein receptor (SNARE) synaptosomal-associated protein 25 (SNAP-25)^9,10^, which is essential for mediating vesicular fusion and exocytosis. Cleavage of SNAP-25 leads to inhibition of neurotransmitter/neuropeptide release, including acetylcholine, from neuronal cells and is responsible for BoNT/A’s observed pharmacological effects on smooth and skeletal muscles and glands^1,11,12^.

Initially, BoNT/A binds with relatively low affinity (K_D_ ~ 200 nM) to gangliosides, including GT1b, which are abundant at the presynaptic membrane and serve to trap BoNT/A within the extracellular matrix^13–16^. Based on previous work by Rummel and colleagues, who identified the key role of the H_C_/A double mutants (W1266L;Y1267S) in ganglioside binding^17^, these H_C_/A mutations have been demonstrated to disrupt binding to GT1b and, likely, additional gangliosides (eg, GD1a, GD1b, GQ1b, and GM1), which bind to BoNT/A with lower affinity compared to GT1b^14–16^. Once anchored close to the membrane, BoNT/A interacts with one or more relatively high-affinity protein receptors, including the synaptic vesicle protein, SV2 (K_D_ ~ 100 nM)^18–23^, and potentially fibroblast growth factor receptor 3 (FGFR3) (K_D_ ~ 15 nM)^24,25^. Mutations within H_C_/A that affect binding to SV2 have been identified, including T1145A;T1146A (which reduces binding)^20^, and G1292R^19^ (which strongly disrupts binding). The observed in vivo selectivity of BoNT/A for specific classes of neuronal cells is likely due to avidity upon binding to multiple receptors, which may also serve as a requirement to trigger internalization into the neuronal cell via endocytosis^13–16^. Interaction of BoNT/A with multiple receptors could provide an evolutionary advantage for the toxin, since it decreases the likelihood of host-specific mutations, resulting in toxin resistance. A similar strategy is known from other pathogens, including herpes simplex virus (HSV)^26^, *Trypanosoma cruzi* (Chaga’s disease)^27,28^, and human immunodeficiency virus (HIV)^29,30^. Influenza A virus infection has also been suggested to involve FGFRs^31^. Previous protein complex immunoprecipitation results demonstrated an interaction between FGFRs and SV2 in neuronal cells^24^, suggesting the possibility of a multi-receptor BoNT/A complex.

FGFR3 is one of four receptor-tyrosine kinases (FGFR1–4) that act as receptors for fibroblast growth factors (FGFs). FGFR1–3, but not FGFR4, exist in three different splice variants, a–c, which differ in their extracellular ligand binding domains, each with differing ligand binding affinities and specificities^32–34^. The a isoform variants terminate early, resulting in a secreted extracellular FGF-binding protein^35–38^. The b isoform variants, including FGFR2b, are primarily expressed in tissues of epithelial (surface tissue) origin, while the c isoform variants, including FGFR1c and FGFR3c, are primarily expressed in tissues of mesenchymal (connective tissue) origin. Native ligands for FGFRs are generally produced by either epithelial or mesenchymal cells and act on opposite tissue type FGFRs. An exception is FGF1, which binds to both b and c FGFR isoforms^39,40^. There are 22 known FGF ligands that bind with different affinity and selectivity to the different FGFR splice variants. For example, FGF4 binds to FGFR1c > 2c > 3c, while FGF9 binds to FGFR3c > 2c > 1c and 3b, and FGF10 binds to FGFR2b > 1b^36,38,40,41^. Most FGFs interact locally with FGFRs in a paracrine or autocrine manner, although a number of FGFs, including FGF19, FGF21, and FGF23, act like hormones in an endocrine manner^37,42^. Selectivity and affinity in vivo is achieved via interactions with co-receptors, including: heparin, heparan sulfate (HS), neural cell adhesion molecule (NCAM), cadherin, integrin, Klotho, anosmin, neuropilin, and fibronectin, which interact with both FGFs and FGFRs^43,44^. Gangliosides have also been reported to be co-receptors for FGFRs, affecting ligand binding, receptor dimerization, receptor activity, and subcellular localization^45,46^.

This tissue-specific expression of the ligands, receptors, and co-receptors guides tissue and organ growth and development. FGFR signaling upon ligand binding and receptor activation is associated with numerous cellular functions, including development, homeostasis, and metabolism. FGFRs are activated by dimerization induced by co-receptor and ligand binding, which enables the cytoplasmic kinase domains to transphosphorylate one another at specific tyrosine residues^35–38,47^.

As noted above, the binding domain of botulinum neurotoxin, rH_C_/A (recombinant form), has been shown to bind FGFR3 in vitro and in cells^24^. To further study wild-type and mutant variants of rH_C_/A interactions with FGFRs and compare these to native FGF ligand interactions, a novel receptor dimerization cell-based assay was developed. Total internal reflection fluorescence (TIRF) microscopy was combined with fluorescence correlation spectroscopy (FCS) and number and brightness (N&B) analysis to develop an assay, hereafter referred to as the “FCS & TIRF receptor dimerization assay,” that measures fluorescently tagged receptor dimerization in live transfected cells. By combining the two methods, the FCS & TIRF receptor dimerization assay allows monitoring of receptor dimerization at the cell membrane in real-time—in the presence of co-receptors, in any cell type, without the need to create a custom reporter cell line or reliance on the use of functional reporters, eg, receptor phosphorylation sites or down-stream kinases.

In the study presented here, the FCS & TIRF receptor dimerization assay was used to evaluate FGFR dimerization in the absence or presence of wild-type and mutant variants of rH_C_/A in transfected neuronal-like (PC-12) cells. PC-12 cells are known to be sensitive to BoNT/A, and addition of GT1b to the media further increases BoNT/A sensitivity^48–50^. To validate the method, FGFR dimerization with native FGFs possessing known receptor selectivity was evaluated in parallel. In addition, the impact of ganglioside and SV2 receptor interactions on rH_C_/A-mediated FGFR dimerization was studied. To better understand the specificity of rH_C_/A-induced dimerization of FGFRs, dimerization with another tyrosine kinase growth factor receptor, epidermal growth factor receptor (EGFR), was also evaluated. Our results demonstrate that rH_C_/A, in the presence of GT1b, induces dimerization of FGFRs with a preference towards FGFR3c, further supporting a functional role of FGFRs in BoNT/A cellular binding, as previously suggested^24^.

## Results

### FCS & TIRF receptor dimerization assay for studying receptor dimerization in live cells

**Figure 1:**
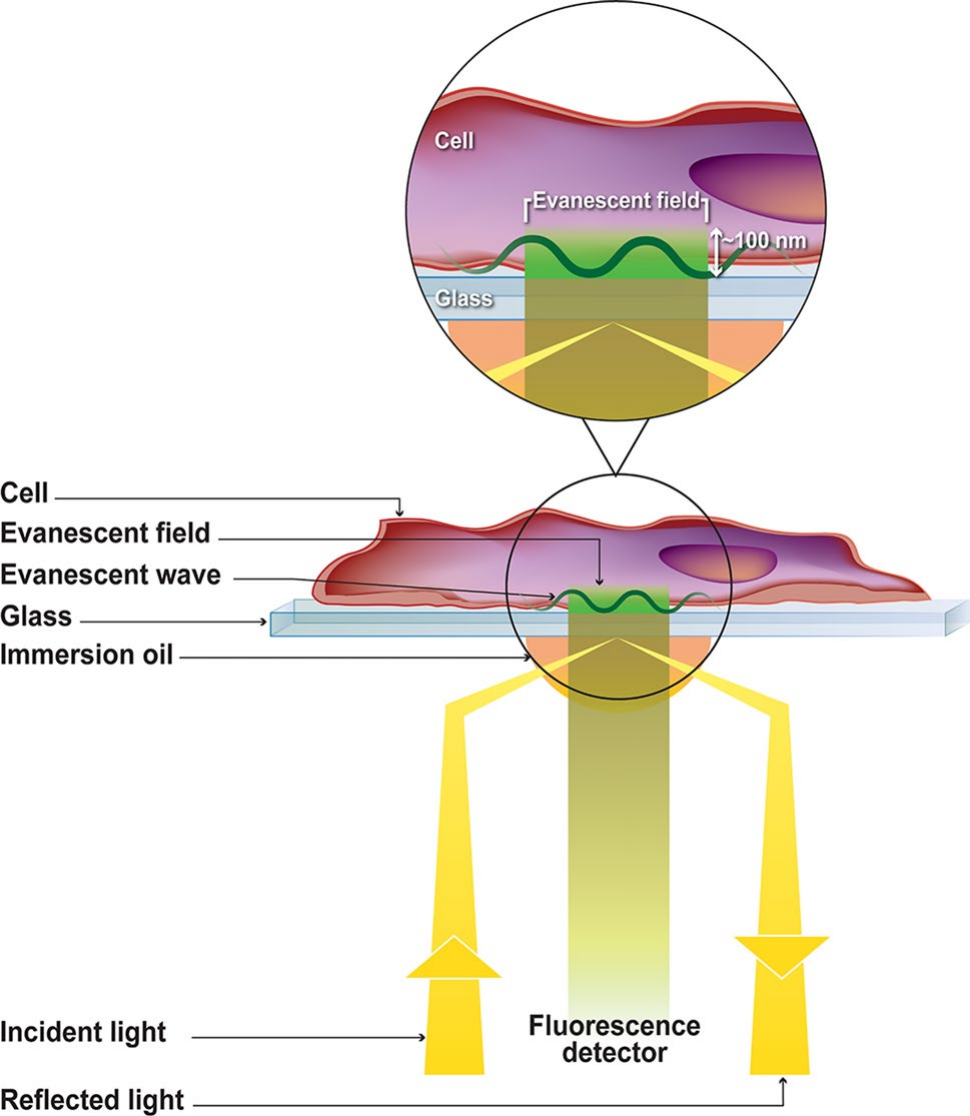
Illustration of the total internal reflection fluorescence (TIRF) method. Adapted with permission from Jameson DM. *Introduction to fluorescence*. Taylor & Francis, 2014.

**Figure 2:**
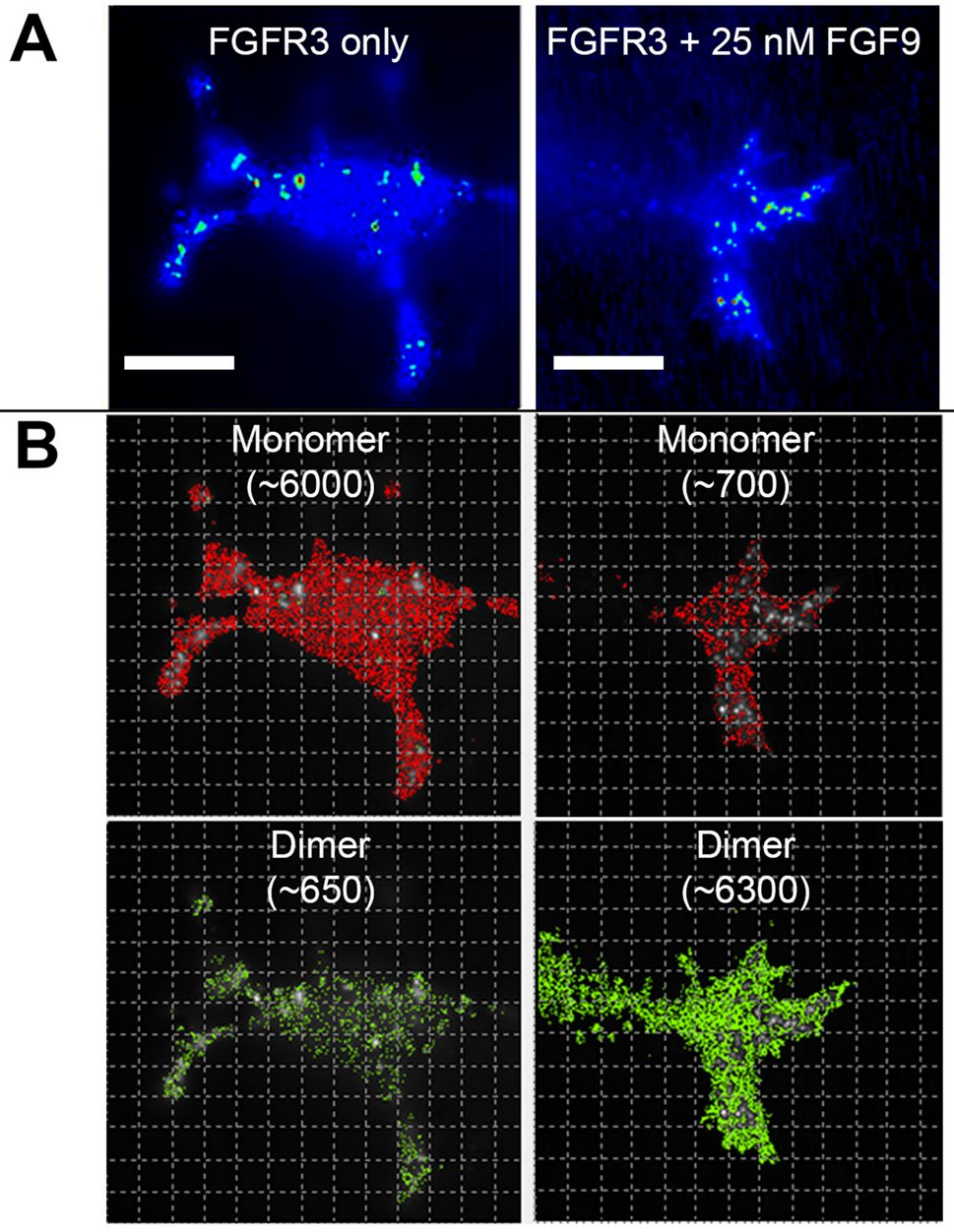
Depiction of intensity and molecular brightness images. **(A)** (Intensity) Typical single cell intensity images are shown for cells that have not been exposed to FGF9 ligand (left column) and cells exposed to 25 nM FGF9 ligand (right column). Scale bar is 15 microns. (**B)** (Molecular brightness) The normalized average molecular brightness images for each cell indicate monomer (red) and dimer (green) numbers and distribution throughout the cell with corresponding pixel counts, which is the number of pixels that contain dimer or monomer, respectively.

In the TIRF (Total Internal Reflection Fluorescence) method, the optics of the instrument are adjusted such that the exciting light will be reflected from the interface, i.e., the glass surface supporting the cell. However, some of the energy of the incident beam will penetrate through the interface, creating what is termed an evanescent field, which extends a very short distance, on the order of 100 nm, into the cell. Hence, this evanescent beam will only be able to excite fluorophores that are located near the cell surface—i.e., the plasma membrane of the target cell (Fig. 1)^51^. Fluorescence fluctuation spectroscopy and number and brightness (N&B) analysis is an emerging method for analysis of molecular interactions in living cells and is based on the statistical analysis of signal fluctuations emitted by fluorescently labeled molecules^51–62^ (see Supplementary Fig. S1 online). The method utilizes the fact that fluorescently labeled proteins produce intensity fluctuations as they pass through a small observation volume. Since the method relies on movement of fluorescent molecules, only mobile molecules are evaluated. While the average intensity in two sample volumes containing the same number of fluorophores may be the same, the fluctuations in intensity depend on the molecular brightness of the fluorescent protein molecules in the samples, and the magnitude of the fluctuations contains information about molecule concentration and the oligomeric state. By measuring the average fluorescence intensity within each pixel (photon counts per second per molecule), the average oligomeric state within each pixel is determined by calculating the molecular brightness—which is directly related to the stoichiometry of fluorophores in a protein complex—and normalizing it to a molecular brightness monomer standard. In this case free fluorophore (AF-488) was used as the monomer standard. For example, if a fluorescently labeled monomeric FGFR is found to homodimerize under addition of a ligand, a twofold increase in the average molecular brightness would be observed compared to the monomeric brightness standard, and the number of pixels with twofold normalized average molecular brightness would increase (Fig. 2B and see Supplementary Fig. S2 online).

Using the FCS & TIRF receptor dimerization assay, the ratio of monomer to dimer fluorescent FGFRs on the plasma membrane of transfected PC-12 cells was measured, with and without addition of FGF ligand or rH_C_/A protein. PC-12 cells are known to be sensitive to BoNT/A, and exogenous GT1b was added to further increase the availability of potential co-receptors. Cells were transfected with FGFR1c, FGFR2b, and FGFR3c, which represents mesenchymal, epithelial, and neuronal FGFRs, respectively^32,34,63^. Briefly, PC-12 cells were transiently transfected with FGFRs containing a C-terminal HaloTag and subsequently fluorescently labeled with HaloTag ligand AF488^53^. TIRF intensity measurements of transfected PC-12 cells indicated a non-homogenous distribution of fluorescence (Fig. 2A), perhaps indicative of aggregated receptor proteins. Immobile fluorophores do not fluctuate, so any potentially aggregated protein will not be analyzed. Calculation of the average molecular brightness, in the absence of ligand, showed that the values for FGFR3c-AF488 were higher (1.1–1.2-fold) than the Alexa488 fluorophore molecular brightness monomer standard, suggesting that 10–20% of the receptors are in preformed dimers. This observation is consistent with literature suggesting that FGFRs and EGFR can dimerize in the absence of ligand^64,65^. Since FGFR expression levels have been reported to affect dimerization, images were collected only from single cells with similar level of transfected receptor expression, an emission of 10,000–15,000 counts per cell, corresponding to approximately 100,000–150,000 receptors per cell, based on the Alexa488 fluorophore monomer standard. Typical intensity images corresponding to monomer and dimer populations are shown in Fig. 2, where saturation with native ligand (FGF9 for FGFR3c) caused  an approximate twofold increase in normalized average molecular brightness, consistent with transition to a 100% dimerized state.

### rH_C_/A induces FGFR3c dimerization

**Figure 3:**
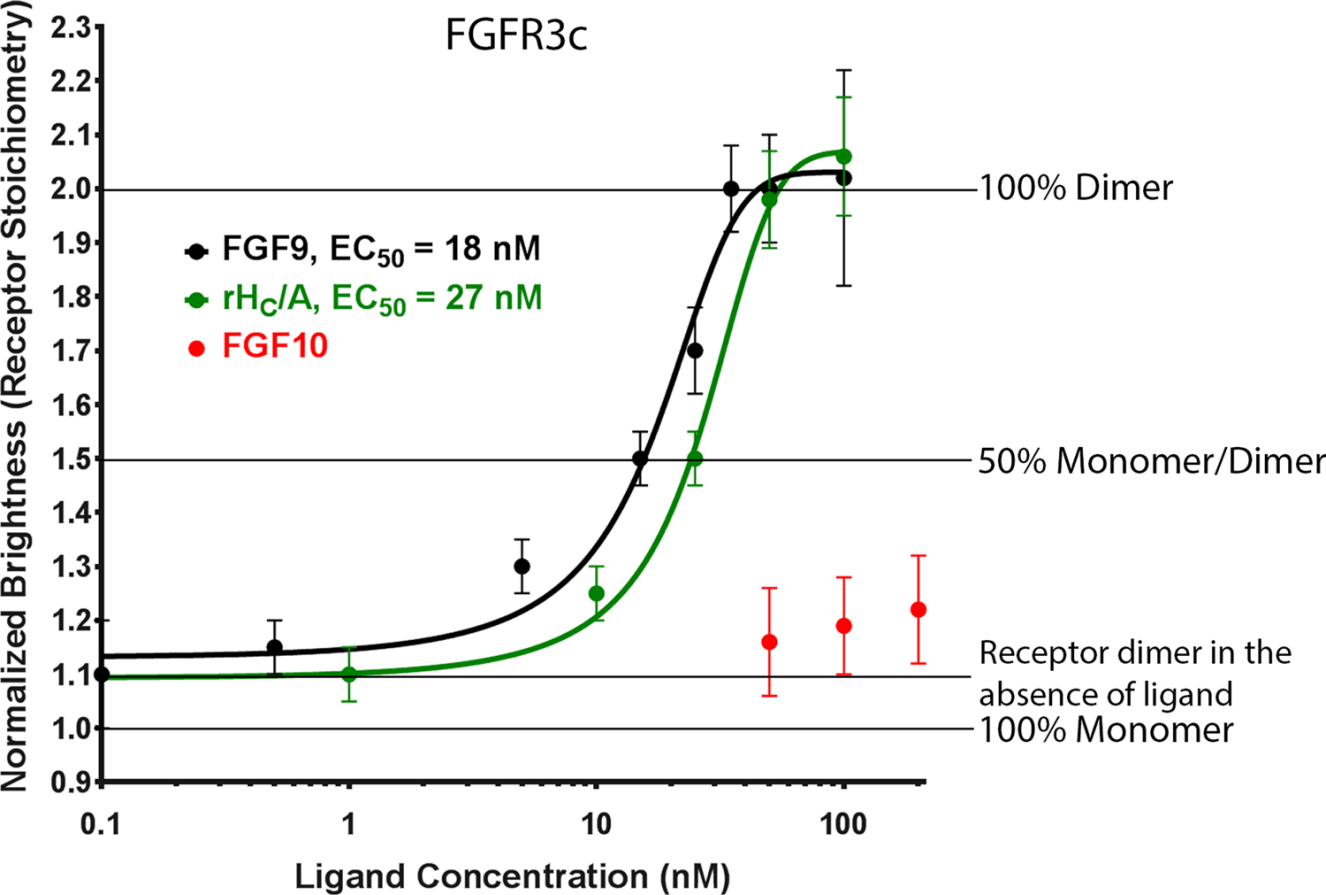
rH_C_/A induces FGFR3c dimerization. Native ligand FGF9 (black) (EC_50_ = 18 nM [95% CI; 13, 24]) and BoNT/A binding domain (rH_C_/A) (green) (EC_50_ = 27 nM [95% CI; 18, 41]), but not FGF10 (red), dimerize fluorescently tagged FGFR3c in transfected PC-12 cells. Points represent the average normalized brightness values ± SD from greater than 30 cells collected on 4 independent days.

Previously, rH_C_/A was shown to bind to extracellular loops 2 and 3 of FGFR3 in vitro with a K_D_ of ~ 15 nM^24^, which is similar to the affinity of native FGFs^40^. To validate the FCS & TIRF receptor dimerization assay and to compare the ability of FGFs and rH_C_/A to dimerize FGFR3c, PC-12 cells transfected with FGFR3c-AF488 were treated with FGF9, FGF10, and rH_C_/A in parallel. FGF9 is a known agonist ligand for FGFR3c and FGF10 is a known agonist ligand for FGFR2b but not FGFR3c. As expected, addition of FGF9 resulted in a dose-dependent increase in average normalized molecular brightness, indicative of receptor dimerization, whereas treatment with FGF10 did not. Addition of rH_C_/A resulted in receptor dimerization, similar to FGF9. EC_50_, defined as the concentration of ligand required for dimerization of 50% of the receptors, was 18 nM (95% CI; 13, 24) for FGF9 and 27 nM (95% CI; 18, 41) for rH_C_/A, suggesting that both act as ligands for FGFR3c (Fig. 3 and Table 1).

**Table 1:** FCS & TIRF receptor dimerization assay—results summary.

Receptor	Ligand	GT1b	Function	EC_50_ (nM)	95% CI^b^	R^2^	*p*-value for Runs test
FGFR1c	FGF4	Yes	Native FGFR1c ligand	17	(8, 35)	0.9689	0.7000
	FGF10		Native FGFR2b ligand	N/A^a^	N/A	N/A	N/A
	rH_C_/A		BoNT/A binding domain	163	(131, 202)^c^	0.9952	> 0.9999
FGFR2b	FGF10		Native FGFR2b ligand	19	(13, 26)	0.9949	0.5429
	FGF4		Native FGFR1c ligand	N/A^a^	N/A	N/A	N/A
	rH_C_/A		BoNT/A binding domain	70	(59, 82)	0.9963	0.8000
FGFR3c	FGF9		Native FGFR3c ligand	18	(13, 24)	0.9664	0.4286
	FGF10		Native FGFR2b ligand	N/A^a^	N/A	N/A	N/A
	rH_C_/A		BoNT/A binding domain	27	(18, 41)	0.9804	0.9000
	rH_C_/A	No	BoNT/A binding domain	44	(36, 54)	0.9971	> 0.9999
	rH_C_/A W1266L;Y1267S	Yes	BoNT/A binding domain, ganglioside mutant	143	(127, 161)	0.9997	0.9000
	rH_C_/A T1145A;T1146A		BoNT/A binding domain, SV2 mutant	78	(53, 115)	0.9956	0.9000
EGFR	EGF		Native EGFR ligand	14	(11, 19)	0.9793	0.7000
	rH_C_/A		BoNT/A binding domain	N/A^a^	N/A	N/A	N/A

^a^No binding observed at highest dose of ligand tested.

^b^Based on 3PL with bottom > 1, top = 2.

^c^Based on 2PL with bottom = 1, top = 2.

### rH_C_/A-induced dimerization of FGFR3c is augmented by GT1b ganglioside

**Figure 4:**
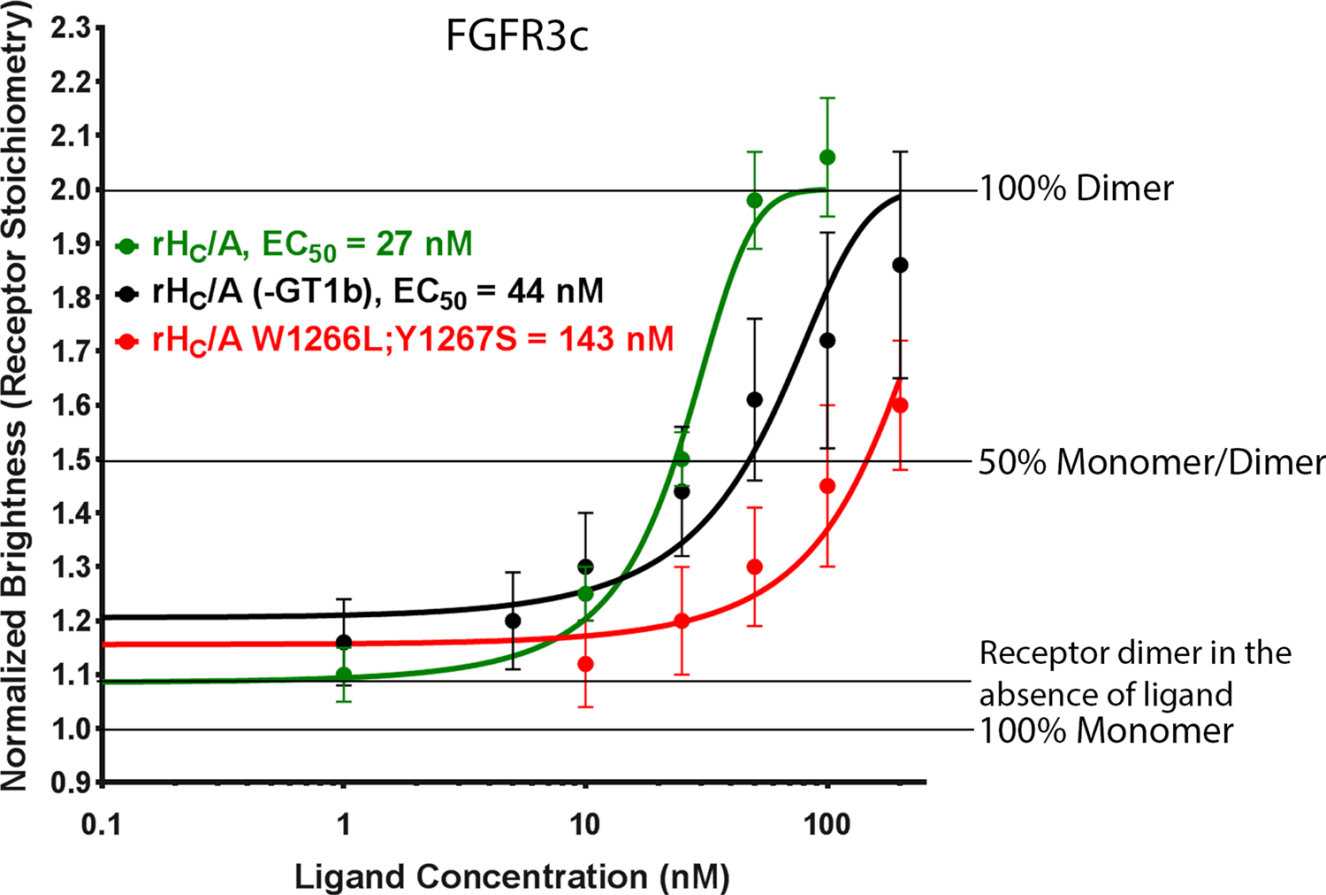
GT1b augments rH_C_/A-induced FGFR3c dimerization. BoNT/A binding domain (rH_C_/A) is more effective at dimerizing fluorescently tagged FGFR3c in the presence of exogenous ganglioside (GT1b) (green vs black, EC_50_ = 27 nM [95% CI; 18, 41] vs EC_50_ = 44 nM [95% CI; 36, 54]), and a BoNT/A binding domain (rH_C_/A) ganglioside mutant variant (W1266L;Y1267S) has further reduced ability to dimerize FGFR3c (red) (EC_50_ = 143 nM [95% CI; 127, 161]). Points represent the average normalized brightness values ± SD from greater than 30 cells collected on 4 independent days.

Gangliosides, such as GT1b, serve as abundant, low-affinity receptors for BoNT/A on neuronal cells^13–16^. Since gangliosides can also function as co-receptors for FGFRs^45,46^, the FCS & TIRF receptor dimerization assay was performed with and without addition of exogenous GT1b to assess its potential role in rH_C_/A-induced fluorescently tagged FGFR3c dimerization. The H_C_/A mutant lacking ganglioside binding (rH_C_/A W1266L; Y1267S^16^) was also tested to assess potential interactions with exogenous and endogenous gangliosides. Previously, it was shown that rH_C_/A W1266L;Y1267S protein lost its ability to bind to neuronal cells, but maintained its ability to bind and cross epithelial barriers, and that antibodies raised against rH_C_/A W1266L;Y1267S protected against full-length BoNT/A at the neuromuscular junction^16^, suggesting that the protein was folded and stable. In the absence of exogenous GT1b, rH_C_/A showed reduced ability to increase molecular brightness, indicative of reduced FGFR3c dimerization compared to dimerization in the presence of GT1b; EC_50_ = 44 nM (95% CI; 36, 54) vs EC_50_ = 27 nM (95% CI; 18, 41) (overlapping 95% CI), respectively. Cells treated with rH_C_/A W1266L;Y1267S in the presence of exogenous GT1b showed further decreased ability to induce receptor dimerization (EC_50_ = 143 nM (95% CI; 127, 161) (Fig. 4 and Table 1). The fact that dimerization of FGFR3c by rH_C_/A, in the absence of exogenous GT1b, was higher than dimerization by rH_C_/A W1266L;Y1267S, in the presence of exogenous GT1b, suggests that rH_C_/A dimerization of fluorescently tagged FGFR3c involves endogenously expressed gangliosides. These results suggest that GT1b, and likely additional gangliosides present on the cell surface of PC-12 cells, augments rH_C_/A-induced FGFR3c dimerization.

### rH_C_/A dimerizes FGFRs in the rank order FGFR3c > FGFR2b > FGFR1c

**Figure 5:**
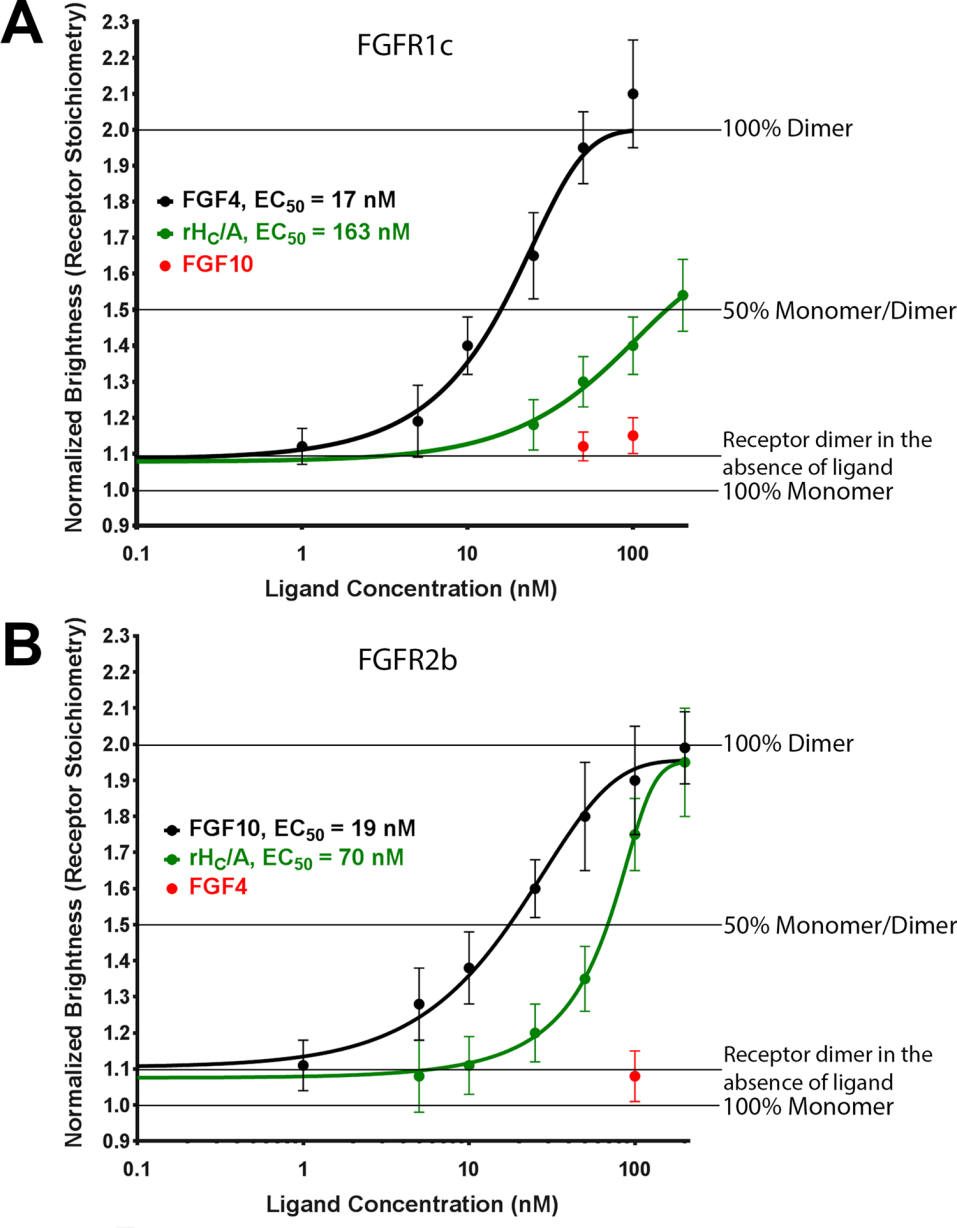
FGFR binding preference of rH_C_/A. **(A)** BoNT/A binding domain (rHC/A) (green) dimerizes fluorescently tagged FGFR1c with reduced potency compared to fluorescently tagged FGFR3c, EC_50_ 163 nM (95% CI; 131, 202)* vs 27 nM (95% CI; 18, 41) **(Fig. ****3****)**. A known native FGFR1c ligand, FGF4 (black) (positive control), dimerizes FGFR1c, EC_50_ = 17 nM [95% CI; 8, 35], while a known native FGFR2b ligand, FGF10 (red), does not. Points represent the average normalized brightness values ± SD from greater than 20 cells collected on 4 independent days. **(B)** BoNT/A binding domain (rH_C_/A) (green) dimerizes fluorescently tagged FGFR2b with reduced potency compared to fluorescently tagged FGFR3c, EC_50_ 70 nM (95% CI; 59, 82) vs 27 nM (95% CI; 18, 41) (Fig. 3), respectively. A known native FGFR2b ligand, FGF10 (black) (positive control), dimerizes FGFR2b, EC_50_ = 19 nM (95% CI; 13, 26), while a known native FGFR1c ligand, FGF4 (red), does not. Points represent the average normalized brightness values ± SD from greater than 30 cells collected on 4 independent days. *Based on 2PL with bottom = 1, top = 2.

As the FGFR subtypes (FGFR1–4) share a high degree of structural and functional homology, it was of interest to assess the ability of rH_C_/A to dimerize other fluorescently tagged FGFRs, including FGFR1c and FGFR2b (Fig. 5 and Table 1). As expected, ligands known to bind specifically to these receptors (ie, FGF4/FGFR1c and FGF10/FGFR2b) caused an increase in molecular brightness—indicative of receptor dimerization—with 100% dimerization appearing around 50–100 nM. The EC_50_ for FGF4-induced FGFR1c dimerization and FGF10-induced FGFR2b dimerization were 17 nM (95% CI; 8, 35) and 19 nM (95% CI; 13, 26), respectively. No increase in dimerization was observed with the non-binding receptor ligand pairings FGF10/FGFR1c and FGF4/FGFR2b. Addition of rH_C_/A resulted in a dose-dependent increase in both FGFR1c and FGFR2b dimerization, but with lower potency compared to the native ligands and compared to rH_C_/A-induced FGFR3c dimerization. The EC_50_ for rH_C_/A-induced FGFR1c and FGFR2b dimerization were 163 nM (95% CI; 131, 202) and 70 nM (95% CI; 59, 82), respectively.

### Mutations in the rH_C_/A SV2 binding site affects FGFR3c dimerization

**Figure 6:**
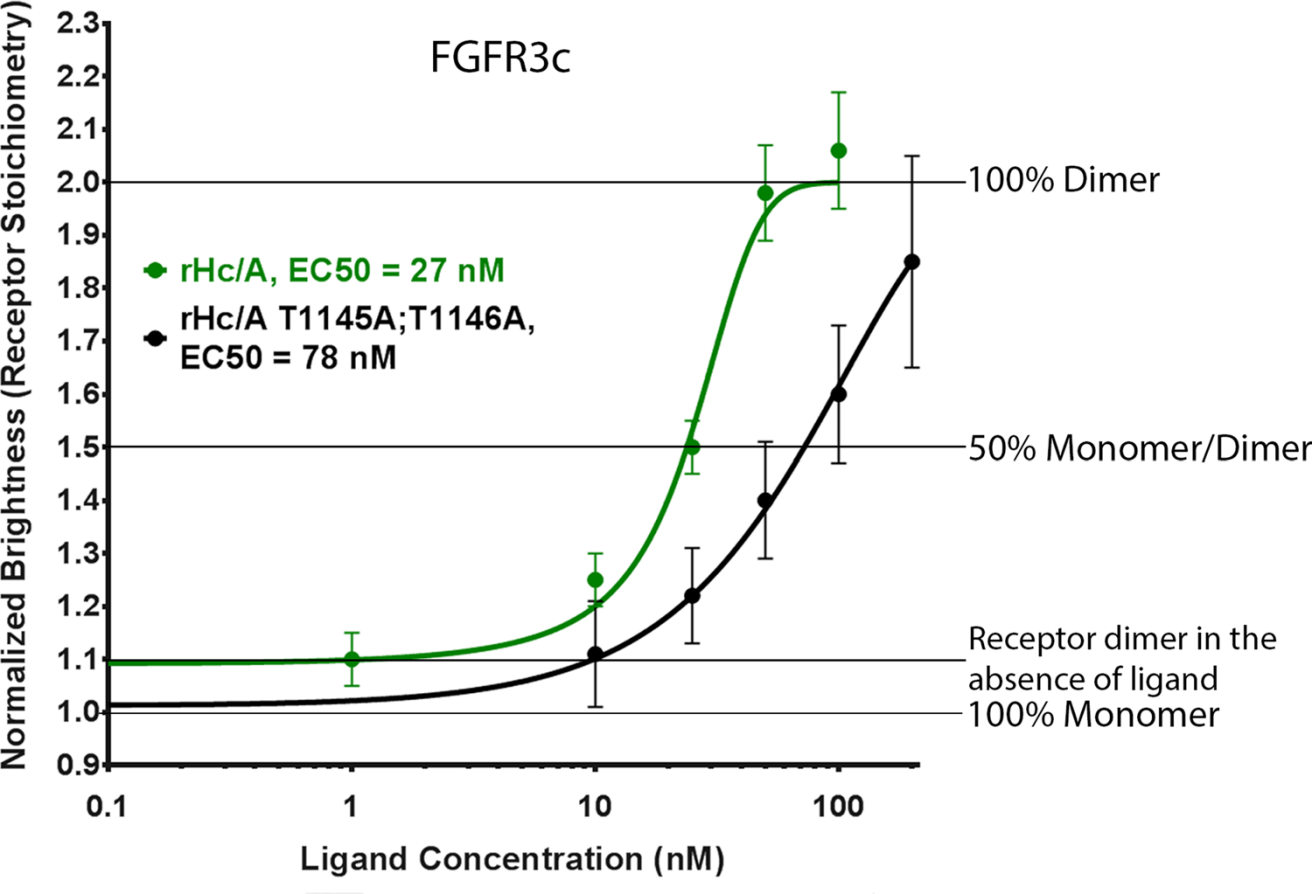
Mutations in the rH_C_/A SV2 binding site affects FGFR3c dimerization. A BoNT/A binding domain SV2 mutant variant (rH_C_/A T1145A;T1146A) (black) has reduced ability to dimerize FGFR3c compared to a wild-type BoNT/A binding domain (rH_C_/A) (green), EC_50_ = 78 nM (95% CI; 53, 115) vs 27 nM (95% CI; 18, 41). Points represent the average normalized brightness values ± SD from greater than 30 cells collected on 4 independent days.

BoNT/A’s interaction with SV2 has been well characterized and mapped to the fourth luminal domain of SV2 and a beta-strand within H_C_/A^19,20^. As previous results suggested that FGFR and SV2 interact in cells^24^, it was of interest to assess the potential role of SV2 binding for rH_C_/A-induced fluorescently tagged FGFR3c dimerization. Addition of an SV2 mutant variant of H_C_/A (rH_C_/A T1145A;T1146A^20^) resulted in a dose-dependent increase in molecular brightness, indicative of receptor dimerization, but with reduced potency (EC_50_ = 78 nM [95% CI; 53, 115]) compared to wt rH_C_/A (EC_50_ = 27 nM [95% CI; 18, 41]) (Fig. 6 and Table 1). Similar to the effect observed without GT1b, these data suggest that residues in H_C_/A that interact with SV2 also affect dimerization of FGFRs, either directly or indirectly. It is unclear how the T1145A;T1146A mutations affect the folding of rH_C_/A. Previously, it was shown that rH_C_/A T1145A;T1146A bound with lower affinity to a recombinant SV2C luminal domain in vitro^20^. The fact that it still binds, albeit with reduced affinity, suggests that the protein is folded similarly to the wild-type protein.

### rH_C_/A does not dimerize EGFR

To assess the selectivity of rH_C_/A for FGFRs versus similar growth factor receptors, the ability of rH_C_/A to dimerize fluorescently tagged EGFR was assessed. EGFR is another tyrosine kinase growth factor receptor that shares structural and functional homology with FGFRs. The native ligand is EGF^66–68^. As expected, addition of EGF resulted in a dose-dependent increase in molecular brightness, indicative of EGF receptor dimerization (EC_50_ = 14 nM [95% CI; 11, 19]). No increase in dimerization was observed in the presence of rH_C_/A (Table 1 and see Supplementary Fig. S2 online), suggesting that rH_C_/A has a high degree of specificity for FGFRs.

## Discussion

In the current study, wild-type and mutant variants of rH_C_/A were evaluated in parallel with native FGF ligands possessing known FGFR subtype selectivity to assess rH_C_/A’s ability to induce fluorescently tagged FGFR receptor dimerization in transfected PC-12 cells. This assessment was done using an FCS & TIRF receptor dimerization assay, a method for measuring molecular associations between proteins by quantifying the molecular brightness of a fluorophore determined through fluctuations in the intensity (due to protein diffusion) within each pixel^56,57^. This method allows for receptor dynamics to be examined in live cells in response to various ligands, providing continual observation under alternating conditions. The focus of the current investigation was to study FGFR dimerization. However, for future studies, this method can potentially provide detailed information on ligand-receptor stoichiometry, cluster formation, and kinetics of receptor endocytosis through the use of fluorescently labeled ligands. The advantage of the FCS & TIRF receptor dimerization assay is that it allows real-time analysis of receptor interactions in live cells as well as direct assessment of the spatial organization of receptor self-association state(s) on the plasma membrane. This approach presents a new method for addressing biological questions not accessible using conventional terminal endpoint approaches.

Utilizing the FCS & TIRF receptor dimerization assay, dimerization of FGFRs and EGFR were monitored in live neuronal cells in the presence of native and novel receptor ligands, such as rH_C_/A. Consistent with other reports^65^, low level of FGFR dimerization (10% to 20%) was observed in the absence of receptor ligand, independent of whether exogenous gangliosides were added or not. The analysis was done with cells expressing approximately 100,000–150,000 tagged FGF receptors per cell to minimize potential effects associated with different densities of receptor on the plasma membrane. The observed increase in molecular brightness with increased concentration of ligand was due to dimerization rather than polymerization. This conclusion follows from the fact that the assay exclusively measures the population of receptors with normalized brightness values indicative of monomer or dimer states (Fig. 2). The fact that a plateau of normalized brightness at ~ 2 (or dimer species) was observed for the non-clustering molecular brightness may indicate that these cells have been saturated with ligand to the point that the endocytosis machinery cannot recycle the receptors fast enough to clear dimeric complexes within the assay timeframe.

The EC_50_ values for FGF ligands dimerizing their cognate fluorescently tagged receptors ranged from 17 to 19 nM (95% CI; 8, 35), which is consistent with what has been reported previously for FGFR ligand binding and signaling^40^, while the EC_50_ values for rH_C_/A were in the rank order FGFR3c (27 nM [95% CI; 18, 41]) > FGFR2b (70 nM [95% CI; 59, 82]) > FGFR1c (163 nM [95% CI; 131, 202]), suggesting that BoNT/A has a preference for the dimerization of the FGFR3c receptor subtype. The cellular expression for each receptor subtype was similar (10,000–15,000 counts per cell) and the EC_50_ value for the native ligands was similar for different FGFR subtypes, while the EC_50_ value for H_C_/A differed. Keeping in mind that an artificial system is utilized, these results support a model where FGFR is part of the specific binding and, potentially, internalization of BoNT/A in neuronal cells. Whether dimerization of FGFRs by BoNT/A could result in pharmacological effects in vivo and in patients is unclear, and it should be kept in mind that clinical toxin doses are in the pM range. Interestingly, unlike most FGFs, which are specific to either the b or c isoform of the different receptor subtypes, rH_C_/A dimerized both b and c isoforms, suggesting that H_C_/A has the potential to affect a large number of cell types that express FGFRs^39,40,42,69,70^. rH_C_/A did not dimerize EGFR at the highest concentration tested here (150 nM) (EC_50_ > 150 nM), which indicates rH_C_/A’ s specificity for FGFR over other tyrosine kinase growth factor receptors (summary of the results shown in Table 1). Whether FGFRs are dimerized by BoNT/A in other cell types and whether other BoNT serotypes can also dimerize FGFRs remains to be further explored.

The observation that gangliosides increase rH_C_/A-mediated dimerization of FGFR3c, while an rH_C_/A variant in which the residues required for GT1b binding have been mutated (W1266L;Y1267S^16^) showed reduced ability to dimerize FGFR, suggests that GT1b and possibly other gangliosides aid in the dimerization of FGFRs by BoNT/A. FGFRs are known to internalize following dimerization via mechanisms involving interactions with extended synaptotagmin-like protein E-Syt2^71–78^. In our study, the presence of BoNT/A led to the dimerization of FGFR. It is possible that the dimerization of FGFR resulting from interaction with BoNT/A may utilize the same internalization mechanism; however, this speculation requires further investigation.

The specific binding site on H_C_/A for SV2 has been identified as an exposed beta-strand loop in the center of the binding domain^19,20,22,79^. The observation that a variant of H_C_/A (T1145A;T1146A^20^) with mutations in residues important for SV2 binding shows reduced ability to dimerize FGFR3c suggests that these residues, directly or indirectly, affect dimerization of FGFRs. However, it is also possible that the reduced binding affinity observed by the H_C_/A T1145A;T1146A variant may arise due to perturbations adjacent to the FGFR binding region of H_C_/A.

In summary, the data presented here show that the binding domain of BoNT/A, rH_C_/A, at nanomolar concentrations, dimerizes fluorescently labeled FGFRs in transfected neuronal-like PC-12 cells. Although these results are based on the use of a model cell system with transfected receptors, they nevertheless further support a model wherein FGFRs—in particular FGFR3c, which is the primary FGFR subtype expressed in the nervous system^32,34,63^—function as receptors for BoNT/A. The potential pharmacological consequence of BoNT interactions with FGFRs may further illuminate the understanding of BoNT neuronal selectivity, potency, and potential receptor-mediated effects.

## Methods

### Materials

All cell culture medium and transfection reagents were from Thermo Fisher (Carlsbad, CA) unless otherwise stated. FGF proteins (FGF4, FGF9, FGF10) and EGF were from R&D Systems (Minneapolis, MN). Collagen IV coated glass bottom 35 mm imaging dishes were from MatTek (Ashland, MA). Rat pheochromocytoma cells (PC-12) and fetal bovine serum (FBS) were from Sigma-Aldrich (St. Louis, MO). GT1b was from Enzo Life Sciences (Farmingdale, NY). C-HaloTag-FGFRs, which included FGFR1c (EX-Y2820-M50, accession number: NM_023110.2, *Homo sapiens* fibroblast growth factor receptor 1), FGFR2b (EX-Z6888-M50, accession number: NM_001144913.1, *Homo sapiens* fibroblast growth factor receptor 2), and FGFR3c (EX-M0098-M50, accession number: NM_000142.4, *Homo sapiens* fibroblast growth factor receptor 3), and C-HaloTag-EGFR (EX-A8661-M50, accession number: NM_005228.4, *Homo sapiens* epidermal growth factor receptor) constructs were from Genecopoeia (Rockville, MD). AlexaFluor488 Halo ligand (AF-488) was from Promega (Madison, WI). Protease inhibitor cocktail was from Sigma-Aldrich. Benzonase nuclease and rLysozyme were from EMD Sigma (Billerica, MA). HisTRAP FF and DEAE columns were from GE Healthcare (Chicago, IL).

### Cell culture

PC-12 cells (Sigma-Aldrich, cat. no. 88022401-1VL) were cultured on 100 mm collagen IV plates (cat. no. 354453) using complete growth media consisting of RPMI media with 2 mM GlutaMAX, 10% FBS, 10 mM HEPES, 1 mM Sodium Pyruvate, 100 U/ml Penicillin, and 100 µg/ml Streptomycin. Cells were seeded onto imaging dishes and incubated in complete medium supplemented with 25 μg/mL GT1b (unless otherwise noted) until reaching confluency (~ 4 days). The cells were maintained at 37 °C with 5% CO_2_.

### Transfection of cells

PC-12 cells were plated at ~ 1 × 10^6^ cells per 35 mm collagen IV coated glass bottom imaging dish and transfected with a mammalian expression plasmid containing the DNA sequence encoding C-HaloTag-FGFR1c, C-HaloTag-FGFR2b, C-HaloTag-FGFR3c, or C-HaloTag-EGFR. A DNA solution was prepared by adding and mixing (by inversion) 2.5 μg of plasmid with 100 µL OPTI-MEM media. The transfection solution was prepared by adding and mixing (by inversion) 5.0 μL of Lipofectamine 2000 with 100 µL of OPTI-MEM media. Once the DNA and transfection solutions were mixed by inversion and incubated at room temperature for 30 min, the 200 µL aliquot of the lipofectamine/plasmid solution was added to the plate containing the cells. After 6 h of incubation, the medium was replaced with complete medium with or without GT1b and incubated for an additional 24 h prior to addition of the HaloTag ligand.

### Treatment of cells with HaloTag ligand and test compound for cell imaging

After transfection, the cells were incubated with 500 nM AlexaFluor488 Halo ligand for 15 h. The cells were then washed three times (10 min/wash) with fresh complete medium to remove free ligand and incubated in serum-free medium (complete medium minus FBS, plus B-27 and N-2) for 3–4 h prior to imaging.

### Expression and purification of wild-type and mutant variants of H_C_/A

Recombinant H_C_/A (rH_C_/A; spanning aa 876–1296 of full-length BoNT/A1; GenBank accession no. AF48874) was cloned into pET28a + (N-terminal His6-tag). The DNA was codon optimized for expression in *Escherichia coli*. The variants of H_C_/A (W1266L;Y1267S^16^) and H_C_/A (T1145A;T1146A^20^) were made by site-directed mutagenesis (Genewiz, NJ). The variants were expressed and purified as previously described^24^. Briefly, chemically competent BL21(DE3) *E. coli* (Thermo Fisher Scientific, Waltham, MA) were transformed with an rH_C_/A expression plasmid. Cells were grown at 37 °C until OD_600_ reached 0.7 and expression induced with 1 mM Isopropyl β-d-1-thiogalactopyranoside (IPTG). After 16 h of growth at 22 °C, cells were harvested via centrifugation and lysed in buffer containing: 50 mM Tris–HCl pH 8.0, 10 mM EDTA, 100 mM NaCl, 10 mM DTT, 5% (v/v) glycerol, cOmplete EDTA-Free Protease Inhibitor Cocktail (MilliporeSigma, St. Louis, MO), 150 mU/mL rLysozyme, and 50 mU/mL benzonase nuclease, and then sonicated for 5 min. Lysate was cleared through high-speed centrifugation and subjected to Immobilized Metal Ion Affinity Chromatography (IMAC) (HisTrap), buffer exchange (desalting column), and Ion Exchange Chromatography (IEX) using the ÄKTAxpress system (GE Healthcare Bio-Sciences, Pittsburgh, PA). Protein (50 mM HEPES, pH 7.4, 150 mM NaCl) was quantified using a NanoDrop 2000 spectrophotometer (Thermo Fisher Scientific).

### Image acquisition

Images were recorded on a Nikon Eclipse Ti TIRF microscope using a 60X 1.45 NA TIRF oil objective. Images were captured using a cascade 512B EMCCD camera (Photometrics, Tucson, AZ) at 100 frames per second and stopped manually once it had read close to 1000 frames^55,57^. AF-488 was excited at 488 nm (Sapphire SF 488; Coherent, Santa Clara, CA) with less than 30% laser power using an excitation filter cube (488/10 nm bandpass and 500 nm long pass emission filter; Chroma, Bellows Falls, VT) within the infinity space. Intensity images were recorded for each transfected HaloTag receptor in the absence of FGF or rH_C_/A protein ligand at both the beginning and end of each experiment from two separate dishes. The cells were then treated for 30 min with increasing concentration of ligand, reconstituted in PBS containing 0.1% Bovine Serum Albumin at a final concentration of 0.1 to 200 nM, prior to recording TIRF intensity images. Dark counts for the EMCCD camera were recorded at the beginning and end of each experiment. Free fluorophore (AF-488) (90 nM) in serum-free medium was used as the monomeric standard. Images were processed using the N&B function on the software platform SimFCS. Using FCS, the brightness of a particle as well as the number of particles in a given volume (Number and Brightness Analysis, S1) were separately obtained to determine the degree of aggregation of proteins in solution.

The average normalized brightness value ± standard deviation (SD) for each concentration of ligand was calculated based on at least 20 individual cells on 3–4 independent days. The results were plotted using GraphPad 7.02 Prism and fitted to a two- or three-parameter logistic curve (2PL or 3PL) using the log of the concentration to estimate the EC_50_ values and the 95% confidence intervals (95% CI). For the fitting, the curves were constrained with bottom > 1 (3PL) or = 1 (2PL) and the top = 2, reflecting a transition from monomer to dimer state. To evaluate the goodness of the fit, R^2^ values and the *p*-value for the run-test were calculated and reported. For graphical presentation, the concentrations are plotted on a log scale.

### FCS and TIRF receptor dimerization assay

This dimerization assay provides a histogram of fluorescent particles per pixel, with each particle equivalent to self-association state (either as a monomer or dimer). Final calculations of each state were quantified by fitting the fluorescent intensity histogram of particles centered at the monomer and dimer states. The populations (percentages) of each state were calculated from these values.

## Supplementary Information

Supplementary Figures.

## Data Availability

The datasets generated during and/or analyzed during the current study are available from the corresponding author on reasonable request.
