# Supplementary material for: Characterization of clostridium botulinum neurotoxin serotype A (BoNT/A) and fibroblast growth factor receptor interactions using novel receptor dimerization assay
Source: Sci Rep. 2021 Apr 9;11:7832. doi: 10.1038/s41598-021-87331-7 (PMC8035261; doi:10.1038/s41598-021-87331-7)
Supplement: Supplementary file 1 — Supplementary Figures. [file 41598_2021_87331_MOESM1_ESM.pdf]

Supplementary Information

Characterization of clostridium botulinum neurotoxin serotype A (BoNT/A) and fibroblast growth factor receptor interactions using novel receptor dimerization assay

Nicholas G. James, Shiazah Malik, Bethany J. Sanstrum, Catherine Rheaume, Ron S. Broide, David M. Jameson, Amy Brideau-Andersen, and Birgitte Jacky

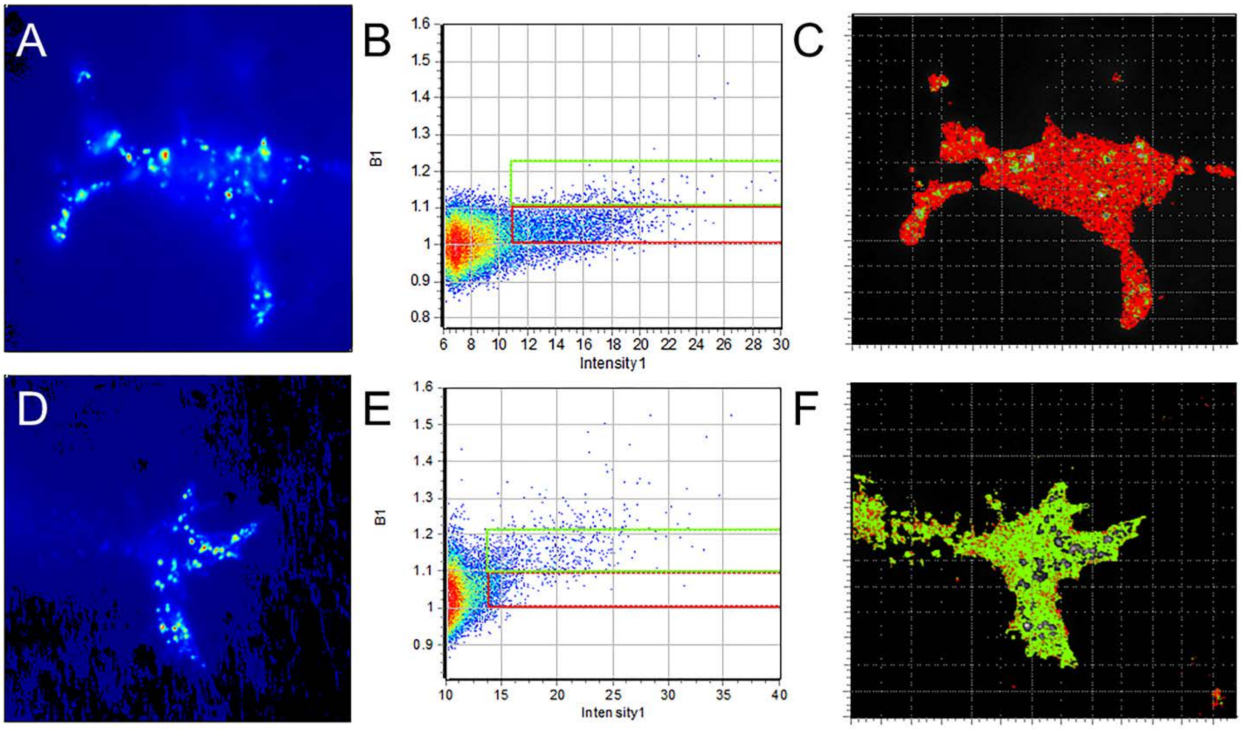

Fig S1. Schematic representation of TIRF N&B analysis of halo tagged FGFR in PC-12 cells. **(A, D)** Intensity images of ~ 1000 images per cell were collected on cells under **(A)** serum starved conditions followed by treatment with **(D)** growth factor. **(B, E)** The intensity fluctuations within each pixel of the collected images are used to calculate the average intensity and brightness in a 2-D histogram. Regions of interest are selected to determine the population of the monomers

(red) and dimers (green). These different populations are represented as an overlay in the images **(C, F)**.

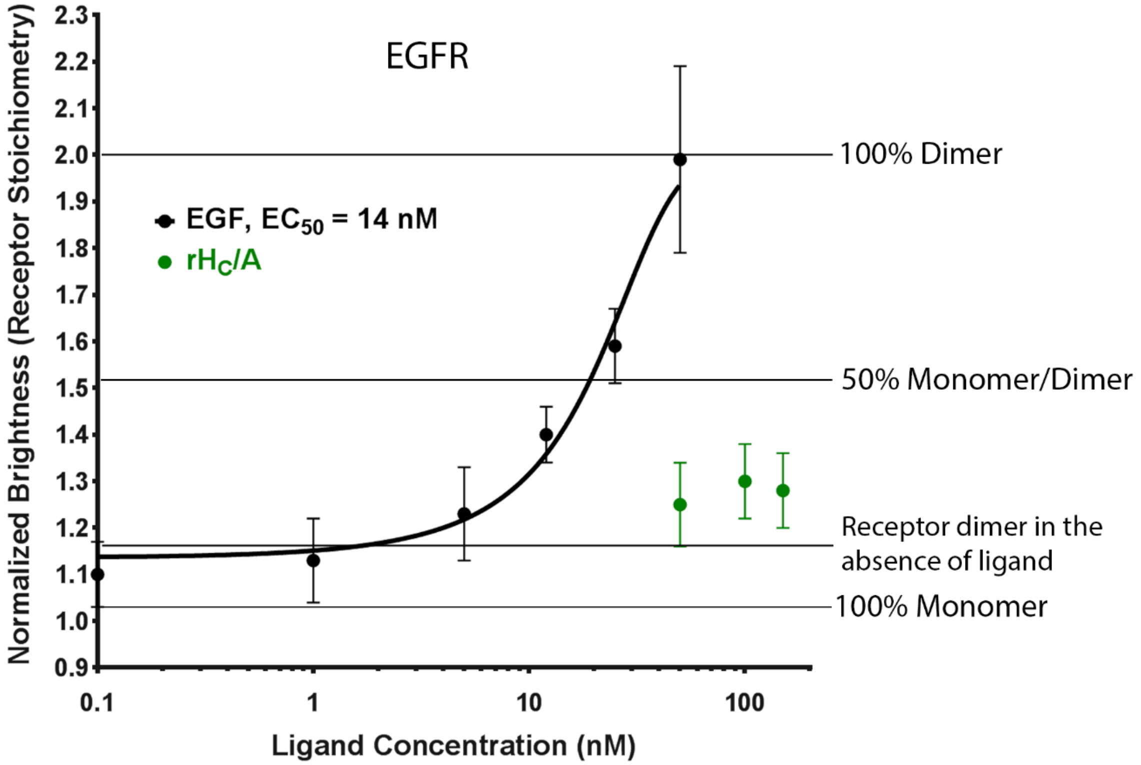

Fig S2. rH_C/A does not induce EGFR dimerization. BoNT/A binding domain (rH_C/A) (green) does not dimerize EGFR (EC_{50} >150 nM), while the native ligand for EGFR, EGF (black) can dimerize with high potency (EC_{50} = 14 nM (95% CI; 11, 19]). Points represent the average normalized brightness values ±SD from greater than 30 cells collected on 4 different days.
